# Correlation of peripheral blood *BMAL1* level with non-motor symptoms in Parkinson’s disease patients

**DOI:** 10.3389/fneur.2026.1962167

**Published:** 2026-09-28

**Authors:** Yanyan Jiang, Yufei Zhang, Luoqing Dong, Hongcan Zhu

**Affiliations:** Department of Neurology, The First Affiliated Hospital of Zhengzhou University, Zhengzhou, Henan, China

**Keywords:** *BMAL1*, Interleukin-6, melatonin, non-motor symptom, Parkinson’s disease

## Abstract

**Objective:**

This study aimed to quantify *BMAL1* expression in the peripheral blood of patients with Parkinson’s disease (PD) and to evaluate its correlation with non-motor symptoms (NMS). We further assessed whether serum interleukin-6 and melatonin levels were associated with *BMAL1* expression.

**Methods:**

This study included 80 patients with PD and 80 age-and sex-matched healthy controls. General clinical information and scores on multiple NMS scales were collected. Peripheral blood *BMAL1* expression was quantified using real-time quantitative polymerase chain reaction. Serum levels of melatonin and interleukin-6 were measured using enzyme-linked immunosorbent assay. Spearman’s correlation analysis was used to evaluate the associations between *BMAL1* expression and the NMS scores.

**Results:**

Compared to healthy controls, patients with PD exhibited significantly lower peripheral blood *BMAL1* expression levels in the morning (*p* = 0.005). *BMAL1* levels were negatively correlated with the NMS Scale score (*r* = −0.407, *p* < 0.001). Furthermore, higher *BMAL1* levels were associated with better night-time sleep quality and cognitive function, whereas lower *BMAL1* levels correlated with more severe anxiety and depression. A weak positive correlation was observed between *BMAL1* expression and melatonin levels (*r* = 0.267, *p* = 0.017). A significant negative correlation was found between *BMAL1* expression and interleukin-6 level (*r* = −0.603, *p* < 0.001).

**Conclusion:**

In this cross-sectional study, peripheral blood *BMAL1* expression at a single morning time point was lower in patients with PD and correlated with NMS severity, suggesting a potential pathway involving *BMAL1*, melatonin, and inflammatory cytokines in NMS pathogenesis. Future studies with multiple time-point measurements are required to validate these findings.

## 1 Introduction

Parkinson’s disease (PD) is a progressive neurodegenerative disorder that is now viewed as a multisystem condition beyond its primary motor impairments, where non-motor symptoms (NMS) such as cognitive decline, mood disorders, sleep disturbances, and autonomic dysfunction profoundly affect patients’ quality of life (1). The motor and nonmotor manifestations of PD exhibit diurnal fluctuations that change throughout the course of the disease, suggesting that the circadian system may be involved in disease progression. The circadian system influences most physiological processes, including sleep, alertness, and cognitive performance (2). Circadian disruption exacerbated nitrated alpha-synuclein pathology and accelerated the loss of dopaminergic neurons in oligomeric alpha-synuclein-treated mice (3). The circadian rhythm governs the sleep–wake cycle through a complex network of clock genes and sleep deprivation leads to decreased *BMAL1* expression (4). Evidence suggests that circadian dysfunction can contribute to cognitive impairment associated with PD, either by directly affecting cognition or indirectly by altering sleep and alertness (5). Sufficient evidence supports an association between disrupted circadian rhythms and clinical mood disorders. Circadian rhythm disturbance is also a major risk factor and common comorbidity of mood disorders, especially anxiety and depression (6).

At the molecular level, circadian rhythms are maintained by a series of clock genes regulated by a transcriptional/translational feedback loop. The circadian locomotor output cycles kaput and the BMAL1 (brain and muscle ARNT-like 1) genes encode proteins that dimerize to form the CLOCK-BMAL1 complex (7). *BMAL1*, a pivotal component of this oscillator, exerts cell-autonomous effects on dopaminergic neuronal survival in animal models. Neuronal *BMAL1* regulates transcripts related to oxidative phosphorylation and PD, and the loss of neuronal *BMAL1* leads to the death of tyrosine hydroxylase-positive neurons in the substantia nigra pars compacta (8). Moreover, conditional *BMAL1* knockout in the suprachiasmatic nucleus or cortical regions induces anxiety- and depression-like behaviors in mice (6, 9). The deletion of *BMAL1* disrupts the core circadian clock and induces widespread glial activation, neuroinflammation, and oxidative stress in the brain. A putative pathway for *BMAL1’s* regulation of neuroinflammation is its transcriptional control of Rev-erbα. Indeed, deletion of Rev-erbα triggers spontaneous microglial and astrocytic activation, enhances NF-κB signaling, and leads to neuronal injury (10). Although findings from animal models provide indirect clues about the role of *BMAL1* in neurodegeneration, direct evidence of its role in patients with PD remains scarce.

Indeed, recent data suggests that clock genes outside the SCN are involved in fundamental brain processes such as sleep/wakefulness, stress, and memory (11). To date, several studies have directly examined peripheral clock gene expression in patients with PD. The initial findings demonstrated that the relative abundance of *BMAL1* was significantly lower in patients compared with control subjects at 21:00, 00:00, and 06:00. In addition, relative *BMAL1* levels correlate positively with PD severity and sleep quality at 06:00 (12). Another study demonstrated the loss of time-dependent variation in *BMAL1* expression in patients with PD (13). A case–control study showed that the expression levels of *BMAL1*, *CLOCK*, *CRY1*, *PER1*, and *PER2* were significantly decreased in peripheral blood mononuclear cells from patients with PD and may serve as endogenous markers of sleep–wake disturbances in PD (14).

However, studies on the correlation between peripheral blood *BMAL1* expression and NMS in patients with PD are relatively limited. Furthermore, previous research has often focused on a single NMS category, lacking a systematic and comprehensive approach. In the present study, we aimed to investigate the relationship between peripheral blood *BMAL1* expression levels and NMS assessments (sleep, cognition, depressive symptoms, and anxiety) in individuals with PD. Disruption of clock genes accelerates neurodegenerative pathologies. Moreover, neuroinflammatory processes synergize with melatonin deficiency to exacerbate the resulting damage (15). To preliminarily explore this mechanism, we aimed to determine whether Interleukin-6 (IL-6) and melatonin levels are correlated with *BMAL1* expression levels.

## 2 Materials and methods

### 2.1 Participants

This observational study included 80 patients (46 males, 34 females) who were admitted to the Department of Neurology at our hospital and diagnosed with PD between July 1, 2023, and July 1, 2025. Patients were diagnosed with idiopathic PD according to the Movement Disorder Society Clinical Diagnostic Criteria. Eighty age- and sex-matched healthy controls (HCs) without any obvious neurologic disorders were recruited from caregivers accompanying patients during the same hospitalization period. Exclusion criteria, applied identically to both PD patients and HCs, included: (1) other neurological disorders (e.g., secondary Parkinsonism, Parkinson-plus syndromes, etc.); (2) severe systemic internal diseases (e.g., heart failure, liver or kidney failure, infections, active malignancy, etc.); (3) obstructive sleep apnea syndrome; (4) major psychiatric disorders (e.g., schizophrenia, bipolar disorder, etc.); (5) use of antidepressants, hypnotics, and anti-inflammatory drugs within the past month; (6) shift work within the past month. All enrolled participants had complete clinical data and *BMAL1* expression results. This study was approved by the Ethics Committee of the First Affiliated Hospital of Zhengzhou University, and written informed consent was obtained from all participants.

### 2.2 Clinical data collection

We obtained basic demographic and clinical data, including sex, age, age at onset, disease duration, and dopaminergic medication, from each patient with PD. The levodopa equivalent daily dose (LEDD) was calculated using an established dose-equivalence method (16). The post-void residual urine volume was measured using ultrasonography during routine urological examinations, and the values were obtained from official ultrasound reports. The Unified Parkinson’s disease rating scale (UPDRS) and the Hoehn and Yahr (H-Y) stage were used to evaluate both motor and non-motor features during the “off” state. The NMS was assessed using the NMS Scale (NMSS). We evaluated rapid eye movement (REM) sleep behavior disorder (RBD) using an RBD single-question screen (RBDSQ). Based on the RBDSQ, patients with scores greater than 5 were categorized as having probable RBD. To further analyze specific non-motor domains, multiple standardized scales were used for subdomain evaluation: the Parkinson’s Disease Sleep Scale (PDSS) and Pittsburgh Sleep Quality Index (PSQI) for nighttime sleep quality, the Insomnia Severity Index (ISI) for insomnia severity, and the Epworth Sleepiness Scale (ESS) for daytime sleepiness. Based to the total ESS score, patients were divided into two groups: ESS group (ESS ≥ 10 points, *n* = 45) and non-ESS group (ESS < 10 points, *n* = 35). The Mini-Mental State Examination (MMSE) and Montreal Cognitive Assessment (MoCA) were used to evaluate cognitive function. The Hamilton Depression Scale (HAMD) and the Hamilton Anxiety Scale (HAMA) to assess depression and anxiety, respectively. The quality of life was assessed using the Parkinson’s Disease Questionnaire (PDQ-39).

### 2.3 Blood sampling and processing

Fasting peripheral blood samples (3 mL) were collected by direct venipuncture at 6 a.m. on the second day of hospitalization and place in an EDTA tube. During blood sampling, the participants were not strictly shielded from external light. However, when the lights were turned off, the illuminance was below 5 lux. The room temperature was maintained at 21 ± 2 °C. Two milliliters of the sample were transferred to a microcentrifuge tube and centrifuged at 3,000 rpm for 15 min, and the upper serum was collected and placed into clean Eppendorf tubes. The serum was then frozen at −80 °C for melatonin and IL-6 measurements. To the remaining blood sample (1 mL), add 3 mL of TRIzol reagent, mix thoroughly, label the tube, and store it at −80 °C.

### 2.4 Peripheral blood mRNA extraction and quantification

Total RNA was extracted using the Trizol reagent (Life Technologies, Waltham, MA, USA) according to the manufacturer’s instructions. The reverse transcription protocol was as follows: 30 min at 42 °C for polyadenylation and reverse transcription, followed by 5 s at 85 °C for enzyme inactivation. The obtained cDNA was used immediately for polymerase chain reaction (PCR) or stored at −80 °C. *BMAL1* mRNA levels were measured by quantitative real-time PCR (RT-qPCR). The PCR reactions were executed using ABI 7500 Fast real-time PCR system (Applied Biosystems, USA) in a total reaction volume of 20 μL. GAPDH was used as a housekeeping gene. Primers were purchased from Service Bio (Wuhan, China). The primer sequences are listed in Supplementary Table S1. After 95 °C for 30 s, the experimental reaction consisted of 40 cycles of 95 °C for 15 s and 60 °C for 30 s, and the target gene was detected by the fluorescent dye SYBR Green I (TIANGEN, Beijing, China). To verify the specificity of SYBR Green amplification, melting curve analysis was performed for each reaction. A single peak at the expected melting temperature was confirmed for all samples, indicating specific amplification. Expression levels were calculated using the 2^−△△Ct^ method.

### 2.5 Serum melatonin and IL-6 measurement

Serum melatonin concentration was evaluated using a Human Melatonin enzyme-linked immunosorbent assay (ELISA) kit (Servicebio, Wuhan, China) according to the manufacturer’s instructions. The melatonin concentration in the samples was calculated using the standard curve equation, which was generated from the known standard concentrations and their corresponding optical density values. ELISA Kit (Servicebio, Wuhan, China) was used to detect IL-6 levels in patients’ serum according to the manufacturer’s instructions. For each sample, ELISA measurements were performed in duplicate, and the mean values were used for analysis.

### 2.6 Statistical analysis

Statistical analyses and graphing were performed using SPSS (version 31.0; IBM Corp., Armonk, NY, USA) and GraphPad Prism (version 8.0; San Diego, CA, USA). Continuous variables are expressed as mean ± standard deviation, while categorical variables are presented as frequencies (*n*) and percentages (%). The Kolmogorov–Smirnov test was used to assess whether the data were normally distributed. Statistical significance for independent groups with normally distributed data was evaluated using *t*-tests. Specifically, we compared *BMAL1* expression levels between the following groups: (1) patients with PD versus HCs, (2) patients with PD with RBD versus those without RBD, and (3) male patients with PD versus female patients with PD. Categorical variables were compared using the Chi-squared test. Correlations between clinical scores and *BMAL1* mRNA levels were analyzed using Spearman’s correlation coefficients. Partial correlation analyses were performed after adjusting for sex, age, disease duration, and LEDD. Two-sided tests were used, and *p* < 0.05 was considered significant.

## 3 Results

### 3.1 Patient characteristics

Among the 80 patients with PD included in the study, the mean age was 64.76 ± 8.78 years, with 46 (57.5%) males and 34 (42.5%) females. The mean age of the HCs was 65.63 ± 8.40 years, with 43 (53.75%) males and 37 (46.25%) females. There were no significant differences in age or sex between patients with PD and the HCs (*p* > 0.05). The demographic and clinical variables of the participants are shown in Supplementary Table S2.

### 3.2 *BMAL1* expression levels in PD patients and HCs

Compared with the HCs, peripheral blood *BMAL1* expression levels were significantly lower in PD patients (0.83 ± 0.59 vs. 1.03 ± 0.16, *p* = 0.005; Figure 1A). Additionally, patients with RBD demonstrated a significantly lower *BMAL1* level compared with those without RBD (0.64 ± 0.57 vs. 1.01 ± 0.57, *p* = 0.005; Figure 1B). No significant difference in *BMAL1* expression levels was observed between male and female patients with PD (0.91 ± 0.62 vs. 0.74 ± 0.55, *p* = 0.194; Figure 1C).

**Figure 1:**
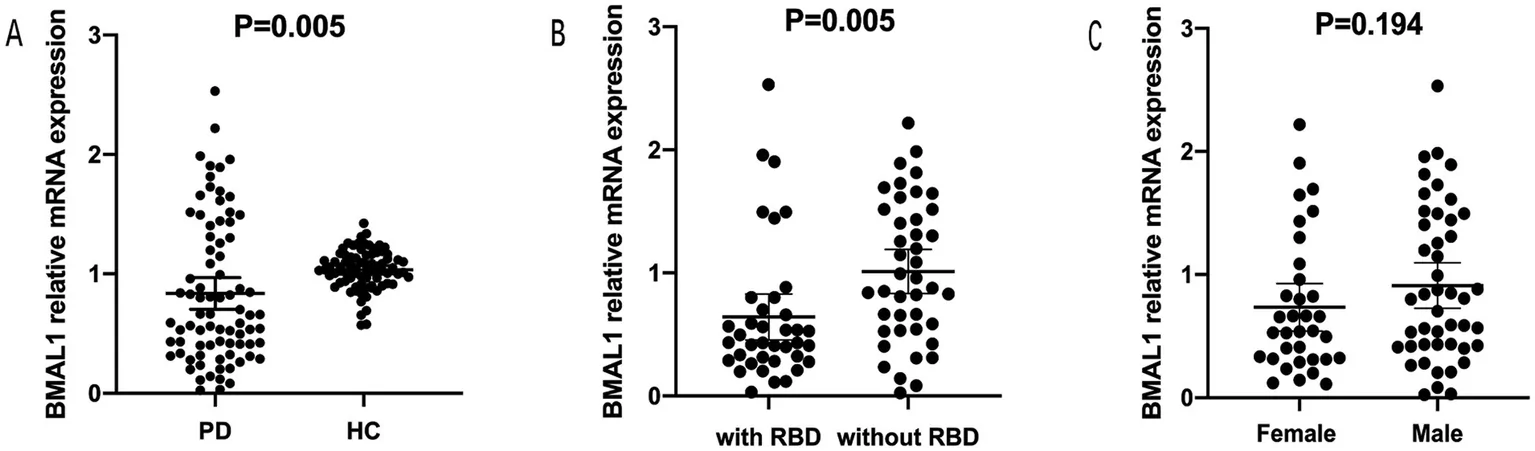
Scatter plots of *BMAL1* relative mRNA expression level in the peripheral blood of HCs and different subgroups of PD patients. **(A)** Comparison between PD patients and HCs. **(B)** Comparison between PD patients with RBD and those without RBD. **(C)** Comparison between male and female PD patients. The data are expressed as the mean/95% confidence interval. PD, Parkinson’s disease; HC: healthy control. RBD: rapid eye movement sleep behavior disorder.

### 3.3 Relationship between *BMAL1* expression levels and NMS in PD patients

Both unadjusted and adjusted correlations among morning *BMAL1* relative mRNA expression, clinical variables, and NMS indicators in patients with PD are presented in Table 1. The partial correlations were adjusted for age, sex, disease duration, and LEDD. The results showed that *BMAL1* level was negatively correlated with the NMSS (*r* = −0.407, *p* < 0.001; Figure 2A) and with the UPDRS-total, part II, and part III scales, but no associations were found with age, age of onset, and disease duration. Partial correlation analysis showed that the *BMAL1* expression levels were positively correlated with MMSE (*r* = 0.373, *p* = 0.001), and MoCA scores (*r* = 0.423, *p* < 0.001; Figures 2B,C), suggesting that patients with higher *BMAL1* levels tend to have better cognitive function. Furthermore, the analysis revealed a significant correlation between *BMAL1* expression and sleep quality. Expression was positively correlated with the PDSS score (*r* = 0.424, *p* < 0.001), whereas negative correlations were observed with the PSQI score (*r* = −0.309, *p* = 0.007) and severity of insomnia ISI score (*r* = −0.331, *p* = 0.004; Figures 2D–F). These results suggest that higher *BMAL1* levels are associated with better nighttime sleep quality. Regarding daytime sleepiness, as assessed by the ESS score, patients in the ESS group had significantly lower *BMAL1* levels (0.62 ± 0.49 vs. 1.03 ± 0.53, *p* = 0.001). Simultaneously, the expression levels of *BMAL1* were negatively correlated with the HAMD (*r* = −0.407, *p* < 0.001) and HAMA scores (*r* = −0.322, *p* = 0.005; Figures 2G,H), indicating that lower *BMAL1* levels were associated with higher anxiety and depression. We found that *BMAL1* expression levels did not correlate with the residual urine volume (*r* = −0.165, *p* = 0.157). There were no significant differences between patients with orthostatic hypotension and without orthostatic hypotension (0.76 ± 0.58 vs. 0.82 ± 0.53, *p* = 0.627). Moreover, *BMAL1* expression levels were negatively correlated with PDQ-39 scores (*r* = −0.500, *p* < 0.001; Figure 2I). Therefore, patients with PD with lower *BMAL1* levels are more likely to experience more severe NMS and lower quality of life.

**Table 1:** Correlation analysis between *BMAL1*relative mRNA level and clinical indicators in PD patients.

Clinical indicators	*BMAL1* level (unadjusted spearman)			*BMAL1* level (partial correlation)^a^		
	*r*	95% CI	*p*	Partial *r*	95% CI	Adjusted *p*
Age	−0.094	−0.313 to 0.135	0.406	–	–	–
Age of onset	−0.087	−0.307 to 0.141	0.441	–	–	–
Disease duration	−0.085	−0.305 to 0.144	0.456	–	–	–
H–Y stage	−0.188	−0.397 to 0.04	0.095	−0.267	−0.499 to −0.002	0.020^*^
UPDRS total score	−0.293	−0.487 to −0.072	0.008^*^	−0.332	−0.547 to −0.058	0.004^*^
UPDRS I	−0.270	−0.468 to −0.047	0.015^*^	−0.219	−0.467 to 0.063	0.059
UPDRS II	−0.346	−0.530 to −0.130	0.002^*^	−0.382	−0.553 to −0.171	<0.001^*^
UPDRS III	−0.246	−0.447 to −0.021	0.028^*^	−0.250	−0.494 to 0.045	0.031
NMSS score	−0.451	−0.615 to −0.251	<0.001^*^	−0.407	−0.579 to −0.243	<0.001^*^
PDSS score	0.428	0.223–0.596	<0.001^*^	0.424	0.252–0.607	<0.001^*^
PSQI score	−0.338	−0.524 to −0.122	0.002^*^	−0.309	−0.518 to −0.112	0.007^*^
ISI score	−0.359	−0.541 to −0.145	0.001^*^	−0.331	−0.535 to −0.133	0.004^*^
MMSE score	0.392	0.182–0.567	<0.001^*^	0.373	0.164–0.568	<0.001^*^
MoCA score	0.449	0.248–0.613	<0.001^*^	0.423	0.225–0.593	<0.001^*^
HAMD score	−0.411	−0.583 to −0.204	<0.001^*^	−0.407	−0.593 to −0.322	<0.001^*^
HAMA score	−0.294	−0.487 to −0.072	0.008^*^	−0.322	−0.479 to −0.140	0.005^*^
RVR	−0.182	−0.392 to 0.046	0.106	−0.165	−0.330 to 0.070	0.157
PDQ-39 score	−0.488	−0.643 to −0.294	<0.001^*^	−0.500	−0.648 to −0.333	<0.001^*^

*r*, correlation coefficient; CI, confidence interval; a, adjusted for age, sex, disease duration, and LEDD. The 95% CIs were generated using bias-corrected bootstrap procedures (1,000 resamples). *p*, *p* value; ^*^p < 0.05. PD, Parkinson’s disease; UPDRS, Unified Parkinson’s disease Rating Scale; NMSS, Non-motor symptoms scale; PDSS, Parkinson disease sleep scale; PSQI, Pittsburgh Sleep Quality Index; ISI, Insomnia Severity Index; MMSE, Mini-Mental State Examination; MoCA, Montreal Cognitive Assessment; HAMD, Hamilton Depression Scale; HAMA, Hamilton Anxiety Scale; RVR, Post-void residual urine volume; PDQ-39, Parkinson’s disease questionnaire. For UPDRS III, the bootstrap 95% CI slightly included zero despite a parametric *p* < 0.05, indicating a marginal association.

**Figure 2:**
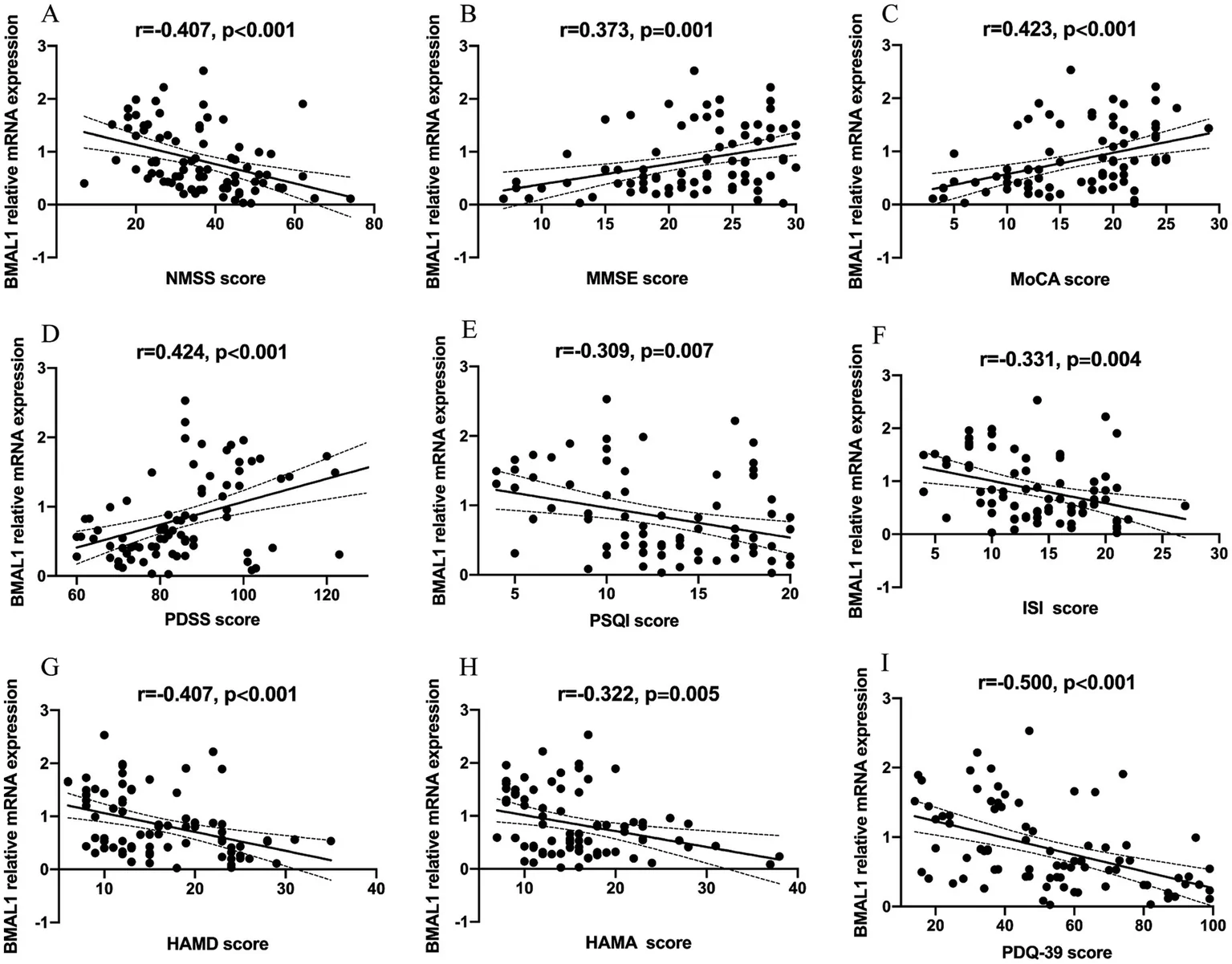
Correlations between *BMAL1* relative mRNA expression level and clinical indicators in PD patients. Scatter plots showing the relationships between *BMAL1* expression and NMSS score **(A)**, MMSE score **(B)**, MoCA score **(C)**, PDSS score **(D)**, PSQI score **(E)**, ISI score **(F)**, HAMD score **(G)**, HAMA score **(H)**, PDQ-39 score **(I)**. *r* and adjusted *p*-values (adjusted for age, sex, disease duration, and LEDD) are shown in each panel. *n* = 80 for all panels. Solid lines represent linear regression fits, and shaded areas indicate 95% confidence bands.

### 3.4 Relationship between *BMAL1* expression levels and melatonin

To gain preliminary insights into this mechanism, we investigated whether a correlation exists between *BMAL1* expression and melatonin levels. A weak positive correlation was observed between *BMAL1* expression and melatonin levels (*r* = 0.267, *p* = 0.017; Figure 3A). We further performed correlations between the clinical variables and melatonin concentrations, and the results are shown in Supplementary Table S3. A positive correlation was observed between serum melatonin levels and PDSS scores, whereas inverse correlations were found with UPDRS Part I, NMSS, PSQI, ISI, HAMD, and HAMA scores (all *p* < 0.05). This pattern indicates a potential link between reduced melatonin levels and increased severity of NMS such as poor sleep quality, anxiety, and depression. The remaining indicators were not associated with serum melatonin levels (*p* > 0.05).

**Figure 3:**
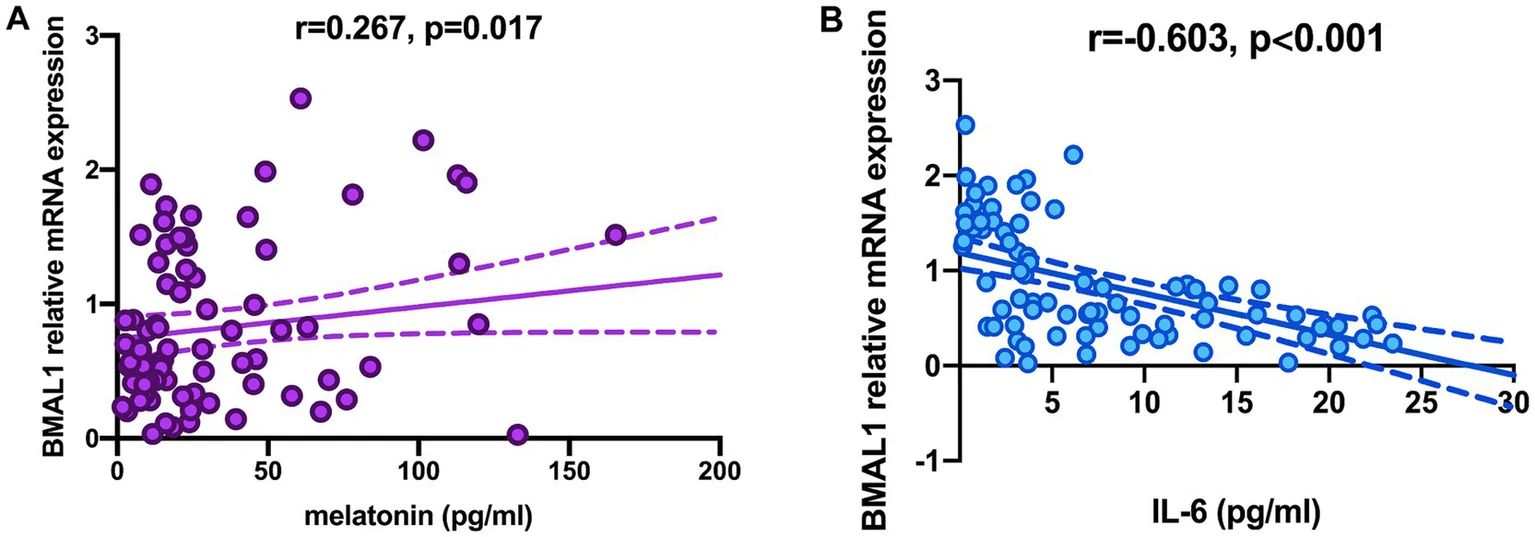
Correlations between peripheral blood *BMAL1* relative mRNA expression level and serum melatonin **(A)** and IL-6 level **(B)** in PD patients (*n* = 80). Solid lines represent linear regression fits, and shaded areas indicate 95% confidence bands.

### 3.5 Relationship between *BMAL1* expression levels and IL-6

We further investigated whether a correlation exists between *BMAL1* expression and inflammatory markers. A significant negative correlation was observed between *BMAL1* expression and IL-6 levels (*r* = −0.603, *p* < 0.001; Figure 3B). Correlation analysis between IL-6 levels and clinical variables is summarized in Supplementary Table S3. IL-6 levels were found to be positively correlated with the UPDRS total score, UPDRS parts I and II, NMSS, PSQI, ISI, HAMD, HAMA, and PDQ-39, but IL-6 levels were inversely correlated with the PDSS, MMSE, and MoCA scores (all *p* < 0.05). Patients with PD who had higher serum IL-6 levels are more likely to experience more severe NMS, including poor sleep quality, anxiety, depression, and cognitive impairment.

## 4 Discussion

Our study systematically evaluated the relationship between peripheral blood core clock gene *BMAL1* expression levels and NMS in patients with PD. The results demonstrated significant correlations between *BMAL1* expression and various NMS, suggesting a potential association between peripheral clock genes and NMS. Our findings are consistent with the hypothesis that *BMAL1* may contribute to the pathophysiology of NMS in PD patients. A recent study demonstrated that more stable circadian rhythms were associated with a reduced burden of NMS, depressive symptoms, and fatigue in individuals with RBD (17). Another animal study indicated that *rhBmal1* knockout disrupts the mammalian circadian clock at both molecular and behavioral levels. Conversely, in a sleep-deprived mouse model, rhBmal1 treatment alleviated anxiety, improved cognitive function, and reduced hippocampal oxidative stress (18). We examined the relationships between clock genes, melatonin, and inflammatory cytokines. Our results revealed significant correlations between *BMAL1* expression and melatonin and IL-6 levels. This raises the possibility of potential biological pathways through which *BMAL1*, melatonin, and inflammatory cytokines may jointly influence NMS. These findings provide preliminary insights into the potential mechanisms underlying NMS. However, these associations should be interpreted as correlational, rather than causal.

In recent decades, the relationship between circadian rhythm genes and sleep disturbances has garnered significant interest. Sleep deprivation results in a significant decrease in the expression levels of the core circadian clock genes, *BMAL1* and *CLOCK* in morning peripheral blood samples (4). In the present study, we observed that patients with RBD had a significantly lower *BMAL1* levels. Previous studies have demonstrated the absence of circadian rhythmicity in the expression of the clock genes *BMAL1* and *Per2* in patients with RBD, implicating altered circadian clock gene expression in the pathophysiology of RBD (19). Our results revealed significantly reduced *BMAL1* levels in patients with ESS. Evaluations of sleep quality using the PDSS, PSQI, and ISI further indicated that *BMAL1* levels may be associated with sleep disturbances in PD. Consistent with our results, a well-conducted study reported alterations in the peripheral molecular clock in PD, suggesting its potential as a biomarker for sleep–wake disturbances in the disease (14). Recent studies have shown that higher morning *BMAL1* expression in patients with anxiety disorder is associated with poorer sleep quality. Specifically, *BMAL1* levels in morning buccal epithelial cells were positively correlated with PSQI indicators (20). One plausible mechanistic explanation is that the altered *BMAL1* levels may reflect or contribute to the neural circuitry governing circadian rhythms, thereby influencing the sleep–wake cycle. Nevertheless, it is plausible that sleep disruption itself leads to the downregulation of *BMAL1* expression.

This study showed that *BMAL1* expression levels were positively correlated with MMSE and MoCA scores, indicating that higher *BMAL1* levels were associated with better cognitive performance. Consistent with the link between circadian dysfunction and cognitive decline, disruption of core clock genes causes memory impairment. Rare *BMAL1* variants have recently been implicated in human neurodevelopmental disorders. Supporting a direct pathogenic role, Drosophila expressing a variant of the circadian clock (*BMAL1* ortholog) recapitulates deficits in short- and long-term memory (21). One study reported dysregulated *BMAL1* expression in AD patients’ brains and demonstrated that elevated CLOCK/BMAL1 activity contributes to astrocyte dysfunction during AD pathogenesis. Another study analyzing DNA methylation at the *BMAL1* locus in the superior frontal gyrus of patients with AD indicates that reduced *BMAL1* activity was correlated with increased AD pathology, particularly tau accumulation and neurofibrillary tangles. Furthermore, the observed positive between *BMAL1* methylation levels and measures of fluid intelligence and vocabulary suggest a critical role for *BMAL1* in supporting cognitive performance, including learning and memory retrieval (22). However, the precise mechanisms by which *BMAL1* directly contributes to memory processes remains unclear.

Circadian rhythm abnormalities can also cause mood disorders. Our study also showed that lower *BMAL1* levels are associated with higher levels of anxiety and depression. A previous animal study demonstrated that mice with SCN-specific knockdown of *BMAL1* exhibited depression-and anxiety-like behaviors, including helplessness and behavioral despair (23). A similar study has showed that *BMAL1*-deficient monkeys exhibit anxiety and depression. The ablation of *BMAL1* upregulates transcriptional programs for inflammatory and stress responses, with transcription networks associated with human sleep deprivation, major depressive disorders, and aging (24). A recent animal study has revealed that dopaminergic neuron-specific *BMAL1* knockout mice exhibit a range of behavioral abnormalities, including hyperactivity, impaired working memory, attention deficits, altered despair-like behavior, and abnormal social behavior (25). In patients with obstructive sleep apnea, morning *BMAL1* expression positively correlated with the severity of depressive symptoms, whereas evening *BMAL1* expression showed no such association (26). Based on these studies, it is hypothesized that *BMAL1* may contribute to anxiety and depression and may indirectly influence emotional states by disrupting sleep. However, reverse causality cannot be excluded: mood disorders themselves are known to disrupt circadian rhythms, and it remains possible that the observed reduction in *BMAL1* levels is a consequence rather than a cause of affective disturbances in PD.

Nocturia is a common but poorly managed symptom in patients with PD. Urine production follows a robust circadian rhythm. The potential contribution of circadian dysregulation to urinary and bladder dysfunction in PD has been overlooked and warrants further study (2). However, no correlation was found between *BMAL1* expression level and residual urine volume. In future research, it is essential to investigate the potential association between peripheral clocks and various autonomic symptoms, using the Scale for Outcomes in PD-Autonomic Dysfunction to evaluate multiple autonomic domains.

Serum IL-6 levels have been reported to correlate with NMS in PD (27). Similarly, we found that IL-6 levels were positively correlated with UPDRS part I, NMSS, PSQI, ISI, HAMD, and HAMA scores, and negatively correlated with PDSS, MMSE, and MoCA scores. To the best of our knowledge, no previous study has focused on the relationship between IL-6 and *BMAL1* expression in patients with PD. Our results revealed that IL-6 is correlated with the expression of the clock gene *BMAL1*. Inflammation is a major modulator of circadian clock function. As the inflammatory regulators control clock gene expression, which is regulated by the circadian clock mechanism, this leads to a vicious cycle of complete circadian misalignment (28). Notably, inflammation and circadian disruption may reciprocally reinforce each other. The present study may increase our understanding of the importance of the circadian regulation of inflammation.

Our study revealed a potential link between reduced melatonin levels and increased NMS severity, including poor sleep quality, anxiety, and depression. Although reduced melatonin levels are consistently associated with poor sleep quality, the causation has not been definitively established. In addition, a weak positive correlation was observed between *BMAL1* expression and melatonin levels. Melatonin has emerged as a promising adjuvant for PD because of its antioxidant properties and regulation of circadian rhythm-related genes (29, 30). A pilot double-blind, crossover, placebo-controlled randomized clinical trial involving 26 patients with PD demonstrated that daily administration of 25 mg of melatonin for 3 months increased morning *BMAL1* levels (31). These findings suggest that melatonin supplementation may contribute to the restoration of circadian function.

This study elucidated the association between *BMAL1* expression levels and NMS in PD and explored the underlying mechanisms, including melatonin dysregulation and neuroinflammation. Notably, understanding the role of circadian dysregulation in PD NMS is complicated by numerous confounding factors, such as medication effects, comorbid primary sleep disorders, and pre-existing psychiatric, cognitive, or autonomic dysfunction (2). Nevertheless, it is important to emphasize that our study precludes causal inference; therefore, the utility of *BMAL1* as a clinical biomarker or therapeutic target requires validation in longitudinal and randomized controlled trials.

This study has some limitations that cannot be ignored. First, the main limitation was the sample size of patients with PD. Second, owing to the cross-sectional study design, no causal relationship between *BMAL1* expression and clinical variables could be established; the reported associations should only be interpreted as correlational. Third, although the central pacemaker is localized to the SCN, we measured clock gene expression in peripheral blood, which has been shown to faithfully reflect patterns in central tissues. Notably, this study analyzed *BMAL1* expression levels at a single time point (at 6 a.m.), which precludes assessing the correlation between *BMAL1* expression rhythms and NMS. Nevertheless, the choice of 6 a.m. was supported by two previous studies (12, 26). *BMAL1* expression at 6 a.m. may be particularly relevant to NMS, thus providing a rationale for focusing on this single time point in the present study. However, this observation warrants further investigation into the dynamics of gene expression across the full 24-h cycle.

## 5 Conclusion

In conclusion, our cross-sectional study demonstrated decreased peripheral blood *BMAL1* expression in patients with PD measured at a single morning time point, and *BMAL1* expression levels were associated with NMS. The results also revealed a correlation between *BMAL1* expression and melatonin and IL-6 levels. This suggests potential biological pathways through which *BMAL1*, melatonin, and inflammatory cytokines jointly influence NMS. Future studies with multiple time-point measurements are required to validate these findings.

## Data Availability

The raw data supporting the conclusions of this article will be made available by the authors, without undue reservation.
