# Supplementary material for: Correlation of peripheral blood BMAL1 level with non-motor symptoms in Parkinson’s disease patients
Source: Front Neurol. 2026 Sep 28;17:1962167. doi: 10.3389/fneur.2026.1962167 (PMC13658855; doi:10.3389/fneur.2026.1962167)
Supplement: Supplementary file 1 [file Table_1.DOCX]

Supplementary Material

# Supplementary Table 1

Table S1 Primer sequences targeting *BMAL1* and GAPDH

| Gene | Sense（5’-3’sequence） | Antisense（5’-3’sequence） |
| --- | --- | --- |
| BMAL1 | CTTCAAGATCCTCAACTACAGCCAG | GTCGTGCTCCAGAACATAATCG |
| GAPDH | GGAAGCTTGTCATCAATGGAAATC | TGATGACCCTTTTGGCTCCC |
