# Supplementary material for: Correlation of peripheral blood BMAL1 level with non-motor symptoms in Parkinson’s disease patients
Source: Front Neurol. 2026 Sep 28;17:1962167. doi: 10.3389/fneur.2026.1962167 (PMC13658855; doi:10.3389/fneur.2026.1962167)
Supplement: Supplementary file 2 [file Table_2.DOCX]

Supplementary Material

# Supplementary Table 2

Table S2 Demographic and clinical variables of the Patients and HCs

| Parameters | PD Patients(n=80) | HCs(n=80) | p |
| --- | --- | --- | --- |
| **Demographics** | | | |
| Age (years), mean ± SD | 64.76±8.78 | 65.63±8.40 | 0.526 |
| Males, mean ± SD | 63.07±8.96 | 65.37±7.95 | 0.204 |
| Females, mean ± SD | 67.06±8.09 | 65.92±9.00 | 0.578 |
| Sex, n (%) |  |  | 0.633 |
| Male | 46 (57.5) | 43 (53.75) | - |
| Female | 34 (42.5) | 37 (46.25) | - |
| **Clinical characteristics** | | | |
| Age of onset (years), mean ± SD | 61.46±8.64 | - | - |
| Disease duration (years), mean ± SD | 3.28±2.27 | - | - |
| Levodopa equivalent daily dose(mg), median (interquartile range) | 400.0 (250) | - | - |
| H–Y stage, median (interquartile range) | 2.0（1.5） | - | - |
| UPDRS total score, mean ± SD | 46.63±16.08 | - | - |
| UPDRS part I, mean ± SD | 6.26±4.24 | - | - |
| UPDRS part II, mean ± SD | 12.79±5.91 | - | - |
| UPDRS part III, mean ± SD | 21.31±8.78 | - | - |
| NMSS score, median (interquartile range) | 36 (19) | - | - |
| RBD, n (%) | 38(47.5) |  | - |
| PDSS score, median (interquartile range) | 85.0(22) | - | - |
| PSQI score, median (interquartile range) | 13 (7) | - | - |
| ISI score, median (interquartile range) | 14 (8) | - | - |
| ESS score ≥10, n (%) | 45(56.25) | - | - |
| MMSE score, median (interquartile range) | 23 (8) | - | - |
| MoCA score, median (interquartile range) | 18.5 (9) | - | - |
| HAMD score, median (interquartile range) | 15 (11) | - | - |
| HAMA score, median (interquartile range) | 15 (8) | - | - |
| Residual urine volume(ml), median (interquartile range) | 7(34) | - | - |
| Orthostatic hypotension, n (%) | 32(40.0) | - | - |
| PDQ-39 score, mean ± SD | 52.92±6.42 | - | - |
| **Biomarkers** | | | |
| *BMAL1* expression, mean ± SD | 0.83±0.59 | 1.03±0.16 | 0.005 |

Data are expressed as mean ± standard deviation (SD) or median (interquartile range).

Abbreviations: H-Y, Hoehn–Yahr; UPDRS, Unified Parkinson’s disease Rating Scale; NMSS, Non-motor symptoms scale; RBD, rapid eye movement sleep behavior disorder; PDSS, Parkinson Disease Sleep Scale; PSQI, Pittsburgh Sleep Quality Index; ISI, Insomnia Severity Index; ESS, Epworth sleepiness scale; MMSE, Mini-Mental State Examination; MoCA, Montreal Cognitive Assessment; HAMD, Hamilton Depression Scale; HAMA, Hamilton Anxiety Scale; PDQ-39, Parkinson's disease questionnaire.
