# Supplementary material for: Correlation of peripheral blood BMAL1 level with non-motor symptoms in Parkinson’s disease patients
Source: Front Neurol. 2026 Sep 28;17:1962167. doi: 10.3389/fneur.2026.1962167 (PMC13658855; doi:10.3389/fneur.2026.1962167)
Supplement: Supplementary file 3 [file Table_3.DOCX]

Supplementary Material

# Supplementary Table 3

Table S3 Correlation analysis between melatonin, IL-6 level, and clinical indicators in PD patients

|  | melatonin level | | | IL-6 level | |
| --- | --- | --- | --- | --- | --- |
| clinical indicators | r | | p | r | p |
| age | 0.115 | 0.308 | | 0.027 | 0.815 |
| Age of onset | 0.114 | 0.316 | | 0.043 | 0.707 |
| disease duration | -0.126 | 0.266 | | -0.069 | 0.542 |
| Hoehn–Yahr stage | -0.144 | 0.203 | | 0.153 | 0.174 |
| UPDRS total score | -0.157 | 0.164 | | 0.239 | 0.033^*^ |
| UPDRS I | -0.305 | 0.006^*^ | | 0.378 | 0.001^*^ |
| UPDRS II | 0.008 | 0.947 | | 0.306 | 0.006^*^ |
| UPDRS III | -0.089 | 0.430 | | 0.093 | 0.411 |
| NMSS score | -0.253 | 0.023^*^ | | 0.284 | 0.011^*^ |
| PDSS score | 0.335 | 0.002^*^ | | -0.378 | 0.001^*^ |
| PSQI score | -0.238 | 0.033^*^ | | 0.255 | 0.022^*^ |
| ISI score | -0.280 | 0.012^*^ | | 0.287 | 0.010^*^ |
| MMSE score | 0.092 | 0.414 | | -0.318 | 0.004^*^ |
| MoCA score | 0.086 | 0.448 | | -0.361 | 0.001^*^ |
| HAMD score | -0.363 | 0.001^*^ | | 0.354 | 0.001^*^ |
| HAMA score | -0.278 | 0.013^*^ | | 0.288 | 0.009^*^ |
| RVR | 0.103 | 0.362 | | 0.097 | 0.394 |
| PDQ-39 score | -0.196 | 0.081 | | 0.375 | 0.001 |

**Abbreviations**: r, Spearman’s correlation coefficient; p, p value; *p < 0.05. PD, Parkinson’s disease; IL-6, Interleukin-6; H-Y, Hoehn–Yahr; UPDRS, Unified Parkinson’s disease Rating Scale; NMSS, Non-motor symptoms scale; PDSS, Parkinson disease sleep scale; PSQI, Pittsburgh Sleep Quality Index; ISI, Insomnia Severity Index; MMSE, Mini-Mental State Examination; MoCA, Montreal Cognitive Assessment; HAMD, Hamilton Depression Scale; HAMA, Hamilton Anxiety Scale; RVR, Post-void residual urine volume; PDQ-39, Parkinson's disease questionnaire.
